# Combining crystallography and EPR: crystal and solution structures of the multidomain cochaperone DnaJ

**DOI:** 10.1107/S0907444913010640

**Published:** 2013-07-19

**Authors:** Thomas R. M. Barends, Richard W. W. Brosi, Andrea Steinmetz, Anna Scherer, Elisabeth Hartmann, Jessica Eschenbach, Thorsten Lorenz, Ralf Seidel, Robert L. Shoeman, Sabine Zimmermann, Robert Bittl, Ilme Schlichting, Jochen Reinstein

**Affiliations:** a MPI for Medical Research, Heidelberg, Germany; b Department of Physics, Freie Universitat Berlin, Berlin, Germany; c MPI for Molecular Physiology, Dortmund, Germany

**Keywords:** molecular chaperones, crystal dehydration, radiation-damage-induced phasing with anomalous scattering, single-wavelength anomalous diffraction, electron paramagnetic resonance, cross-linking, hybrid structure determination

## Abstract

Hsp70 chaperones assist in a large variety of protein-folding processes in the cell. Crucial for these activities is the regulation of Hsp70 by Hsp40 cochaperones. DnaJ, the bacterial homologue of Hsp40, stimulates ATP hydrolysis by DnaK (Hsp70) and thus mediates capture of substrate protein, but is also known to possess chaperone activity of its own. The first structure of a complete functional dimeric DnaJ was determined and the mobility of its individual domains in solution was investigated. Crystal structures of the complete molecular cochaperone DnaJ from *Thermus thermophilus* comprising the J, GF and C-terminal domains and of the J and GF domains alone showed an ordered GF domain interacting with the J domain. Structure-based EPR spin-labelling studies as well as cross-linking results showed the existence of multiple states of DnaJ in solution with different arrangements of the various domains, which has implications for the function of DnaJ.

## Abstract 2

The crystal structure of the N-terminal part of *T. thermophilus* DnaJ unexpectedly showed an ordered GF domain and guided the design of a construct enabling the first structure determination of a complete DnaJ cochaperone molecule. By combining the crystal structures with spin-labelling EPR and cross-linking in solution, a dynamic view of this flexible molecule was developed.

## 1. Introduction   

DnaJ protects the cell from adverse conditions through the prevention of protein aggregation or by assisting the refolding of unfolded proteins (Hendrick *et al.*, 1993 ▶; Hendrick & Hartl, 1993 ▶; Karzai & McMacken, 1996 ▶) and is an essential partner of the chaperone DnaK in bacterial DnaK–DnaJ–GrpE chaperone systems. In eukaryotes, corresponding systems exist in which homologues of the DnaJ proteins, the Hsp40 proteins, function together with Hsp70 proteins, which are the eukary­otic variant of DnaK (Caplan *et al.*, 1993 ▶; Cyr *et al.*, 1994 ▶; Rassow *et al.*, 1995 ▶; Aron *et al.*, 2005 ▶; Qiu *et al.*, 2006 ▶). While sequence homologues of the nucleotide-exchange factor GrpE are missing in eukaryotes, their role is taken over by functional equivalents such as Hop.

In the DnaK ATPase cycle, DnaJ stimulates ATPase activity, causing DnaK to switch from the ATP-bound state which binds substrates weakly to an ADP-bound state which binds substrates tightly. GrpE then stimulates the exchange of ADP for ATP, resetting the cycle. Thus, in performing these ATPase-related functions, DnaJ and GrpE are also deeply involved in regulating substrate binding by DnaK. To this end, their respective activities are finely balanced to achieve optimal chaperone activity (Pierpaoli *et al.*, 1998 ▶).

In contrast to DnaJ from *Escherichia coli* or *Methano­thermobacter thermautotrophicus* ΔH (Laufen *et al.*, 1999 ▶; Popp & Reinstein, 2009 ▶; Russell *et al.*, 1999 ▶), DnaJ from *Thermus thermophilus* stimulates the ATPase activity of DnaK only very weakly (Groemping *et al.*, 2001 ▶; Klostermeier *et al.*, 1999 ▶; Motohashi *et al.*, 1997 ▶). Nevertheless, DnaJ is essential and highly effective in preventing aggregation of challenged protein-folding intermediates as part of the *T. thermophilus* DnaK–DnaJ–GrpE chaperone system.

The prevention of protein aggregation would appear to be especially important and difficult to achieve in organisms living at very high temperatures, such as *T. thermophilus*. Consequently, the *T. thermophilus* DnaK–DnaJ chaperone system has attracted much interest (Motohashi *et al.*, 1994 ▶, 1996 ▶; Klostermeier *et al.*, 1999 ▶; Schlee & Reinstein, 2002 ▶). It has been shown that *T. thermophilus* DnaK and *T. thermophilus* DnaJ combine with the assembly factor DafA to form a DnaK_3_DnaJ_3_DafA_3_ complex, from which DafA is expelled by the substrate protein, which binds to DnaK (Motohashi *et al.*, 1996 ▶; Dumitru *et al.*, 2004 ▶; Watanabe & Yoshida, 2004 ▶). In the presence of DafA, *T. thermophilus* DnaJ reduces substrate binding by DnaK and even replaces substrate bound by DnaK (Klostermeier *et al.*, 1999 ▶).

However, the precise function and mode of action of *T. thermophilus* DnaJ, or of DnaJ in general, is unclear. Structural information could help to remedy this, but such data have been unavailable to date as neither the structure of any full-length DnaJ nor of a domain of *T. thermophilus* DnaJ has been determined. However, structures and sequences of domains of related proteins help to formulate some hypotheses. Like every DnaJ, *T. thermophilus* DnaJ is a multidomain protein which possesses the highly conserved J domain with its characteristic HPD motif (Bork *et al.*, 1992 ▶; Wall *et al.*, 1994 ▶; Szyperski *et al.*, 1994 ▶). *T. thermophilus* DnaJ is a typical type II DnaJ (see Walsh *et al.*, 2004 ▶ for a definition of DnaJ sub­families), *i.e.* it lacks the zinc-finger domains found in type I DnaJs such as that of *E. coli*. Importantly, however, *T. thermophilus* DnaJ can substitute for *E. coli* DnaJ in *in vitro* refolding experiments with the *E. coli* DnaK–DnaJ system, showing that *T. thermophilus* DnaJ is a full representative of the DnaJ family and that zinc-finger domains are not required for cochaperone activity (Groemping *et al.*, 2005 ▶).

In both type II and type I DnaJs the highly conserved J domain is found at the N-terminus. Biochemical studies on the *E. coli* DnaK–DnaJ system have shown that in this system the J domain, and in particular its HPD motif, is important for both DnaK binding and ATPase stimulation (Wall *et al.*, 1994 ▶; Karzai & McMacken, 1996 ▶). Thus, by analogy, in *T. thermophilus* DnaJ the J domain may also serve to interact with DnaK. Directly behind the J domain, *T. thermophilus* DnaJ possesses a polyproline motif consisting of six consecutive proline residues with unknown function. The polyproline motif is followed by a glycine/phenylalanine-rich domain or GF domain as is often observed in DnaJ proteins (Wall *et al.*, 1995 ▶). Because of their high glycine content, the GF domains of DnaJ proteins have sometimes been suggested to serve as a flexible linker between the J domain and the C-terminal domain. However, Karzai & McMacken (1996 ▶) found that both the J and the GF domains are required for DnaK ATPase activation in the *E. coli* system, whereas the J domain on its own was incapable of doing so. Also in *E. coli*, the GF domain was found to be essential for the activation of the ATP-mediated substrate binding of DnaK (Wall *et al.*, 1995 ▶). Moreover, in a eukaryotic system, yeast, the GF domain determines the binding specificity of the Hsp40 Sis1 (Yan & Craig, 1999 ▶). The GF domain is in turn followed by a C-­terminal domain of variable sequence.

The first structural information on a DnaJ domain was obtained from NMR studies of the *E. coli* DnaJ J domain alone and together with the GF domain (Szyperski *et al.*, 1994 ▶; Pellecchia *et al.*, 1996 ▶). While the GF domain was too dis­ordered for an atomic model to be built, the J domain was shown to consist of four helices labelled I–IV, where the N-terminal helix I is connected *via* a short loop to helices II and III, forming a helix–turn–helix motif with the latter two helices in a relative orientation typical of a coiled-coil structure. The HPD motif is positioned in the loop between helices II and III, which was found to be poorly ordered. Helix III is connected to helix IV by a short linker making an ∼90° turn between the two helices. Helix IV is followed by a poorly ordered linker to the GF domain. Studies of a construct with both the J and GF domains showed that although the GF domain was also disordered, the core of this domain is not completely dis­ordered in the *E. coli* protein as indicated by relaxation times. This partial order within the GF domain was proposed to offset the entropic disadvantage of participating in complex formation with DnaK when starting with a completely dis­ordered state with very high entropy (Pellecchia *et al.*, 1996 ▶). Another NMR study of *E. coli* DnaJ domains showed that upon including the GF domain in a construct with the J domain, the dynamics of the J domain change measurably. Including the GF domain in the construct particularly changed the dynamics of the HPD motif aspartate (Huang, Ghose *et al.*, 1999 ▶). The same laboratory also reported that inclusion of the GF domain changes the orientation of helix IV and suggested that the GF domain interacts transiently with the J domain, proposing that this interaction takes place with the surface-exposed area of helix III opposite its interface with helix II (Huang, Flanagan *et al.*, 1999 ▶).

Despite their low sequence identity, the few available structures of C-terminal domains of DnaJs and Hsp40s are remarkably similar (Sha *et al.*, 2000 ▶; Li *et al.*, 2006 ▶; Hu *et al.*, 2008 ▶; Midwest Center for Structural Genomics, unpublished work; Suzuki *et al.*, 2010 ▶), consisting of two β-barrel-like subdomains stacked on top of each other into a rod-like structure 100 Å in length. Two C-terminal domains dimerize by domain swapping involving two helices, resulting in a laundry-pin-like structure, as can be seen in crystal structures of the C-terminal domains of the yeast Hsp40 Sis1 (Li *et al.*, 2006 ▶). Recently, the structure of the monomeric endoplasmic reticulum type III DnaJ protein P58IPK has been reported (Svärd *et al.*, 2011 ▶), which has the J domain at the C-­terminus, following an α-helical N-terminal stalk structurally unrelated to the dimerization domains mentioned above.

Here, we describe an in-depth structural characterization of *T. thermophilus* DnaJ by hybrid methods. Crystals of wild-type *T. thermophilus* DnaJ were grown and their diffraction was improved to 3.8 Å resolution using a dehydration protocol, but they remained resistant to phasing attempts. A divide-and-conquer strategy led us to first determine the structure of a construct containing the N-terminal 114 residues, DnaJTth_114_, comprising the J and GF domains, using radiation-damage-induced phasing with anomalous scattering (RIPAS; Ravelli *et al.*, 2003 ▶; Zwart *et al.*, 2004 ▶; Banumathi *et al.*, 2004 ▶; Ramagopal *et al.*, 2005 ▶). The electron-density map of this structure inspired a third construct, this time of a complete DnaJ from which a disordered stretch of seven amino acids (108–114 in the linker between the GF domain and C-terminal domain; DnaJ_Δ108–114_) was deleted with a view to increasing conformational stability. The subsequent substitution of four residues with methionine (construct DnaJ_Δ108–114/4M_) allowed SeMet-SAD phasing, resulting in the determination of the first crystal structure of a complete functional DnaJ containing J, GF and C-terminal domains to 2.9 Å resolution. This structure was then used as a search model for molecular replacement with data from the wild-type DnaJ crystals, which indicated a large degree of flexibility between the GF and C-terminal domains, which was then investigated by EPR spin-labelling, simple modelling and cross-linking experiments, resulting in a hybrid model of the highly dynamic DnaJ molecule.

## 2. Materials and methods   

### 2.1. Protein expression and purification   

Constructs were prepared by either QuikChange or two-stage PCR. After two-stage PCR, the PCR products were ligated into pCR2.1TOPO (Invitrogen) and positive clones were selected using blue/white screening. Correct clones as identified by sequencing were digested with *Nco*I and *Not*I (New England Biolabs), purified by agarose-gel electrophoresis and ligated into pET24d. For the DnaJ_Δ108–114/4M_ construct, the region coding for residues 108–114 was deleted and residues Leu57, Ile149, Leu180 and Leu233 were mutated to methionine because of their aliphatic nature.

All DnaJ constructs were heterologously expressed in *E. coli* BL21(DE3) cells and were purified essentially as described in Klostermeier *et al.* (1999 ▶) either by ammonium sulfate precipitation and ion-exchange chromatography (DnaJTth_114_) or by a combination of heat denaturation and Ni^2+^-affinity and gel-filtration chromatography (other constructs). DnaJTth_114_ was expressed in *E. coli* BL21(DE3) cells and precipitated from a cell-free extract by adding solid ammonium sulfate to 80% saturation at room temperature, followed by stirring for 30 min at 277 K. The precipitate was recovered by ultracentrifugation (30 min at 277 K at 65 000*g*) and dissolved in buffer *B* (50 m*M* Tris–HCl, 25 m*M* KCl, 10% glycerol, 2 m*M* EDTA, 1 m*M* DTT pH 7.5 at 298 K). Further contaminants were heat-denatured in a water bath at 348 K for 30 min, followed by removal of the precipitated material using ultracentrifugation (30 min, 65 000*g*, 277 K). The supernatant was loaded onto a Q Sepharose column equilibrated with buffer *B*. DnaJTth_114_ was found in the flowthrough and its purity was assessed by SDS–PAGE. When required, the protein was further purified on a Superdex 75 column in buffer *C* (50 m*M* Tris–HCl, 200 m*M* KCl, 10% glycerol, 2 m*M* EDTA, 1 m*M* DTT pH 7.5 at 298 K). The mass of the protein as determined by MALDI–TOF MS was 12 902.7 Da, which is nearly identical to the calculated average molecular mass of the construct minus the N-terminal methionine residue (12 903.4 Da).

For the production of most of the other constructs, *E. coli* BL21(DE3) cells were transformed with the appropriate plasmid and grown on 3–6 l LB medium. For the production of SeMet-DnaJ_Δ108–114/4M_, 5 l minimal medium supplemented with 50 mg l^−1^ l-selenomethionine at 310 K was used (Doublié, 1997 ▶). At an OD_600_ of 0.3–0.8, 1 m*M* IPTG was added and the temperature was lowered to 293 K. After 20 h, the cells were harvested by centrifugation for 10 min at 5000 rev min^−1^ in an SLC-6000 rotor at 277 K. The cells were resuspended in 50 ml buffer *D* (50 m*M* sodium phosphate, 10 m*M* imidazole, 300 m*M* sodium chloride pH 8.0) and a Complete EDTA-free protease-inhibitor cocktail tablet was added. The cells were lysed by two passes through a fluidizer (690 kPa), and the lysate was incubated in a water bath at 343 K for 10 min and then cooled on ice. The mixture was centrifuged for 40 min at 30 000 rev min^−1^ in a Beckman Ti60 ultracentrifuge rotor (277 K) and the supernatant was mixed with 12 ml 50% Ni–NTA agarose in buffer *D*. The resulting suspension was gently agitated for 2 h at 277 K and then poured into a 6 ml column. This column was washed with 60 ml buffer *E* (50 m*M* sodium phosphate, 20 m*M* imidazole, 300 m*M* sodium chloride pH 8.0). The protein was then eluted with 12 ml buffer *F* (50 m*M* sodium phosphate, 500 m*M* imidazole, 300 m*M* sodium chloride pH 8.0). The protein was concentrated by ultrafiltration to 3 ml and submitted to a 340 ml Superdex 200 column equilibrated with buffer *G* (25 m*M* bis-tris, 200 m*M* KCl, 10% glycerol pH 6.5 adjusted with HCl) running at 2 ml min^−1^. Pure fractions as judged by SDS–PAGE analysis were pooled and then washed and concentrated by ultrafiltration in buffer *H* (20 m*M* bis-tris, 25 m*M* KCl pH 6.5 adjusted with HCl) to an *A*_280_^1 cm^ of ∼9–14. The protein was flash-cooled in liquid nitrogen and stored at 193 K.

### 2.2. Crystallization and structure determination   

#### 2.2.1. J/GF-domain construct DnaJTth_114_   

**Figure 1:**
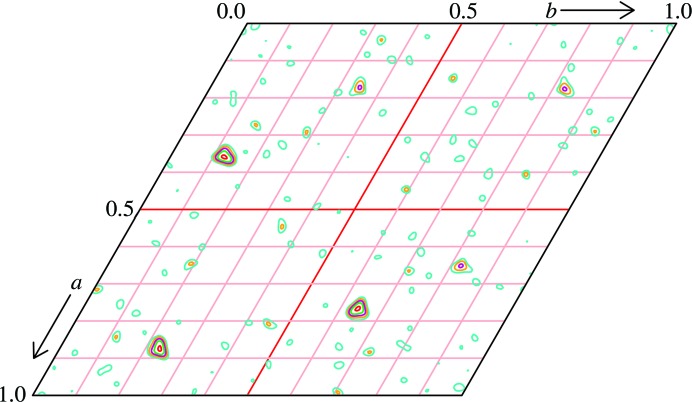
Super-sharpened origin-removed isomorphous difference Patterson section (*w* = 2/3) calculated between the final 120° and the first 120° of data of a 360° data set of the mercury derivative, showing the effect of radiation damage on the mercury derivative. Contour interval = 1σ. This figure was prepared with *XPREP* (Schneider & Sheldrick, 2002 ▶).

DnaJTth_114_ was crystallized by equilibrating hanging drops consisting of 2 µl 17 mg ml^−1^ DnaJTth_114_ in buffer *B* plus 10 m*M* SrCl_2_ and 2 µl 35% PEG 1500 against a 600 µl reservoir of 35% PEG 1500 in Linbro plates. Crystals were cryoprotected in 35% PEG 1500, 10% PEG 400, 25 m*M* Tris–HCl pH 7.5, 10 m*M* SrCl_2_. All data were processed with *XDS* (Kabsch, 1993 ▶) or *DENZO*/*SCALEPACK* (Otwinowski & Minor, 1997 ▶; Otwinowski, 1993 ▶). A mercury derivative was prepared by soaking a crystal in cryoprotectant solution for 16 h which contained 10 m*M* HgCl_2_ instead of SrCl_2_, followed by back-soaking for 1 min. A 360° data set to 2.5 Å resolution was collected on a Rigaku MicroMax-007 HF rotating-anode generator equipped with Osmic mirrors and a MAR345 image plate using Cu *K*α radiation. These data sets showed appreciable anomalous signal, but were nonisomorphous with several other data sets, and SAD phasing was unsuccessful. Since mercury derivatives are prone to radiation damage (Ramagopal *et al.*, 2005 ▶), we set out to phase the data using radiation-damage-induced phasing with anomalous scattering (RIPAS; Ravelli *et al.*, 2003 ▶; Zwart *et al.*, 2004 ▶; Banumathi *et al.*, 2004 ▶; Ramagopal *et al.*, 2005 ▶). The data were reprocessed in two parts (see Table 1 ▶). The first 120° of data were expected to have suffered the least from radiation damage and were used as a derivative. The last 120°, *i.e.* from ϕ = 240° to ϕ = 360°, were expected to have suffered the most from radiation damage and were used as a pseudo-native. Using these data sets, an isomorphous difference Patterson calculated with *XPREP* (Schneider & Sheldrick, 2002 ▶; Fig. 1 ▶) showed peaks in the same positions as in the anomalous difference Patterson map for the whole 360° data set. *SHARP* (Vonrhein *et al.*, 2007 ▶) was used to obtain phases by combining the isomorphous differences between the two data sets with the anomalous difference information, which resulted in an interpretable map (FOM of 0.20 before solvent flattening for all reflections). Using *Xfit* (McRee, 1999 ▶), six copies of the J domain were identified in the electron density. Sixfold averaging and solvent flattening using *RESOLVE* (Terwilliger, 2003 ▶, 2004 ▶) resulted in a much improved map (FOM of 0.61 for all reflections) into which most of the sequence could be built. The final model was obtained through iterative cycles of model building in *Xfit* (McRee, 1999 ▶) and either simulated annealing with *CNS* (Brünger *et al.*, 1998 ▶) or refinement with *REFMAC*5 (Murshudov *et al.*, 2011 ▶), using riding H atoms and TLS refinement, against a high-resolution data set measured on the SLS X10SA beamline at 90 K and a wavelength of 0.9536 Å. Because of the sixfold noncrystallographic symmetry, the reflections used for the calculation of *R*_free_ were selected in thin resolution shells to avoid contamination of the test set. The geometry of the final model was excellent as witnessed by the Ramachandran plot statistics, with 93.9% of the residues in the core regions, 5.9% and 0.2% in the allowed and generously allowed regions, respectively, and 0.0% in disallowed regions. Data-collection and model statistics are given in Table 1 ▶.

#### 2.2.2. DnaJ_Δ108–114/4M_   

Crystals of SeMet-DnaJ_Δ108–114/4M_ were grown by the hanging-drop vapour-diffusion method, equilibrating a drop consisting of 2 µl protein solution with 2 m*M* of various adenine nucleotides and 2 µl reservoir solution against a reservoir consisting of 14–16% PEG 6000, 0.1 *M* succinate/BTP buffer pH 7.0. Crystals were flash-cooled in liquid nitrogen after cryoprotection in 25% ethylene glycol, 16% PEG 6000, 0.1 *M* succinate/BTP buffer pH 7.0 and stored in liquid nitrogen.

A 3.2 Å resolution SAD data set was collected from a crystal grown in the presence of 2 m*M* AMPPNP on the SLS X10SA beamline at a temperature of 90 K and a wavelength of 0.97893 Å. On the same beamline, a 2.9 Å resolution data set was collected at 90 K and a wavelength of 1.00767 Å, *i.e.* at an energy below the Se absorption edge, from a crystal grown in the presence of 2 m*M* ATP-NH_2_. Selenium positions were found using *SHELXD* (Sheldrick, 2008 ▶). Phases were calculated using *autoSHARP* (Vonrhein *et al.*, 2007 ▶), resulting in an interpretable map (phasing power 1.67, FOM_acentric_ = 0.40, FOM_centric_ = 0.13 for the entire resolution range). Inclusion of the 2.9 Å resolution data and solvent flattening with *DM* resulted in an improved map into which the structure was built by iterative cycles of rebuilding using *Coot* (Emsley & Cowtan, 2004 ▶; Emsley *et al.*, 2010 ▶) and refinement with *CNS* (Brünger *et al.*, 1998 ▶) and *REFMAC*5 (Murshudov *et al.*, 2011 ▶). Noncrystallographic symmetry restraints and TLS refinement were used. The anomalous signal present in the SAD data set was used to calculate an anomalous difference Fourier map which was used to locate the Se atoms to guide model building. For the J domains of chains *B* and *D* the 2*mF*_o_ − *DF*_c_ density (Read, 1986 ▶) was poor but recognizable such that a J domain could be placed there. A peak in the anomalous difference electron-density map for the lone Se atom of the labelled J domains confirmed their locations. The final model displayed good geometry, with 91.6% of the residues in the core regions of the Ramachandran plot, 7.8% and 0.6% in the allowed and generously allowed regions, respectively, and 0.0% in dis­allowed regions. Crystal structures and diffraction data have been deposited in the PDB with codes 4j7z (DnaJTth_114_) and 4j80 (DnaJ_Δ108–114/4M_)

#### 2.2.3. Wild-type DnaJ   

Wild-type (WT) DnaJ crystals were produced using the sitting-drop method by equilibrating 2 µl protein solution in buffer *H* with an *A*_280_^1 cm^ of ∼9–14 plus 2 µl reservoir solution and 0.2 µl 24 m*M* CYMAL-5 (Hampton Research, Aliso Viejo, USA) against 600 µl reservoir solution consisting of 1.0 *M* (NH_4_)_2_HPO_4_, 0.1 *M* Tris–HCl pH 8.5. The resulting box-shaped crystals were dehydrated overnight in a saturated solution of trimethylamine oxide (Sigma) in reservoir solution, which served as both a dehydrating agent and a cryoprotectant. Typically, the diffraction resolution improved to around 4 Å after 4 h of dehydration. Dehydration for longer than 20 h reduced the resolution. The best data (3.8 Å resolution) were obtained at the SLS from a crystal which had been soaked for 16 h in 3 m*M* *p*-chloromercuribenzoate in the dehydration solution. The resulting data were phased by molecular replacement with *Phaser* (McCoy *et al.*, 2005 ▶) using a monomer of the C-terminal domain of the SeMet-DnaJ_Δ108–114/4M_ structure as a search model. Only C-terminal domains were observed in the density, and neither molecular replacement nor phased molecular replacement with models constructed from the DnaJTth_114_ structure resulted in local­ization of the J domains. No models were refined.

### 2.3. Spin labelling and EPR measurements   

For spin labelling of DnaJ variants (Todd *et al.*, 1987 ▶, 1989 ▶), the proteins were purified as usual but without a heat-precipitation step in order to minimize cysteine oxidation. Immediately after elution from the Ni–NTA column, the protein was rapidly mixed with a tenfold molar excess of the spin label MTSL [*S*-(2,2,5,5-tetramethyl-2,5-dihydro-1*H*-pyrrol-3-yl)­methyl methanesulfonothioate; Pannier *et al.*, 2000 ▶; Jeschke & Polyhach, 2007 ▶] by rapidly pipetting the protein into a 15 ml tube containing a fivefold molar excess of MTSL dissolved in ∼50 µl acetonitrile while rapidly vortexing, followed by incubation overnight at 277 K. An NAP-25 column (GE Healthcare) was used to remove most of the excess label, after which the purification was continued as usual. After purification, successful labelling was confirmed using MALDI–TOF mass spectrometry. When required, protein samples were deuterated in heavy water using gel filtration and/or ultrafiltration.

Four-pulse ELDOR experiments (also called DEER; double electron–electron resonance; Martin *et al.*, 1998 ▶) were measured at X-band frequencies (around 9.4 GHz) on Bruker Elexsys E580 and E680 spectrometers using an MD5 dielectric ring resonator, Oxford Instruments CF935 cryostats and ITC503 temperature controllers. Experiments on samples in protic buffers were conducted at 80 K. Samples in deuterated buffer were measured at 50 K (single mutants), which allows measurements of longer distances owing to prolonged relaxation times (Jeschke & Polyhach, 2007 ▶). The ELDOR sample DnaJ monomer concentration was between 200 and 280 µ*M*. The pump and detection pulses were set to 32 and 40 ns with a two-step phase cycle for the initial π/2 pulse. The pump pulse was set on the absorption maximum of the nitroxide spectrum and its power was adjusted for maximum inversion efficiency in a two-pulse Hahn echo. Proton modulation was suppressed by an eight-step increase by 8 ns of the initial inter-pulse delay starting at 200 ns. Owing to strong nuclear modulation, this delay was set to 360 ns for the samples in deuterated buffer. The acquired time traces were analyzed using the *MatLab* program *DeerAnalysis* (Jeschke *et al.*, 2006 ▶) employing Tikhonov regularization (Tikhonov & Arsenin, 1977 ▶) after separating the ELDOR signal from background decay owing to isotropic interaction with other surrounding DnaJ molecules at larger distances. Comparison with crystal structures was performed by calculating spin-label rotamer distributions with the program *MMM* (Polyhach *et al.*, 2011 ▶).

### 2.4. Solution cross-linking   

The proteins (5 ml each at concentrations of 170–190 µ*M* as determined by UV absorption at 280 nm using ∊ = 23 505 l mol^−1^ cm^−1^) were dialysed for 16 h against 1 l buffer *I* (25 m*M* HEPES, 25 m*M* KCl, 1 m*M* EDTA pH 7.5 adjusted with NaOH). For cross-linking, fresh proteins were diluted to 10 µ*M* in 30 ml buffer *J* (50 m*M* bis-tris, 10 m*M* EDTA, 100 m*M* NaCl pH 6.5 adjusted with NaOH). To these solutions, an equimolar amount of BMB (bismaleimido­butane; Pierce, Rockford, USA), *i.e.* 1 mol DnaJ dimer:1 mol BMB, was added as follows: 30 µl of 10 m*M* BMB in DMSO was added in ten steps of 3 µl with vigorous stirring at 5 min intervals. After the last addition the solutions were left to incubate for a further 1.5 h while stirring.

To separate DnaJ dimers from DnaJ tetramers generated by interdimer cross-linking, the proteins were concentrated to 100 µl and submitted to a 23 ml Superdex 12 (10/300) column (GE Healthcare) running at 0.8 ml min^−1^ in buffer *K* (50 m*M* HEPES, 200 m*M* NaCl, 2 m*M* DTE). From each mutant, a fraction at the end of the dimer peak (*i.e.* free from tetramer contamination) was investigated using reducing SDS–PAGE to check for the presence of covalently cross-linked DnaJ dimers.

### 2.5. Computational modelling of DnaJ mobility   

Computational modelling of the mobility of DnaJ molecules was performed in *MatLab* using simple models of spherical amino acids. Starting from the crystal structure, an ensemble of conformations was calculated using a Monte Carlo algorithm employing a pseudo-energy term calculated from the EPR distance distributions and the number of clashes between residues. Details are included in Appendix *C* [App appc].

### 2.6. Luciferase-refolding activity measurements   

DnaK luciferase-refolding assays were performed essentially as described in Beinker *et al.* (2002 ▶) and Aponte *et al.* (2010 ▶). Briefly, 10 µ*M* luciferase was de­natured by incubation for 30 min at room temperature in 25 m*M* HEPES pH 7.5, 50 m*M* KCl, 15 m*M* MgCl_2_, 10 m*M* DTE, 0.05 mg ml^−1^ BSA, 1 m*M* ATP with 7 *M* urea. The denatured luciferase was then diluted 125-fold into 25 m*M* HEPES pH 7.5, 50 m*M* KCl, 15 m*M* MgCl_2_, 2 mm DTE, 0.05 mg ml^−1^ BSA, 1 m*M* ATP, 10 m*M* phosphoenolpyruvate, 240 µ*M* coenzyme A, 0.1 m*M* luciferin, 50 µg ml^−1^ pyruvate kinase, to which 3.2 µ*M* DnaK, 0.4 µ*M* GrpE and 0.16, 0.8 or 3.2 µ*M* DnaJ were added. The refolding of luciferase was then followed for 500 min in microtitre plates using the luminescence at 298 K. Details are given in Appendix *A* [App appa].

## 3. Results   

### 3.1. The *T. thermophilus* DnaJ J/GF-domain crystal structure shows an ordered GF domain   

**Figure 2:**
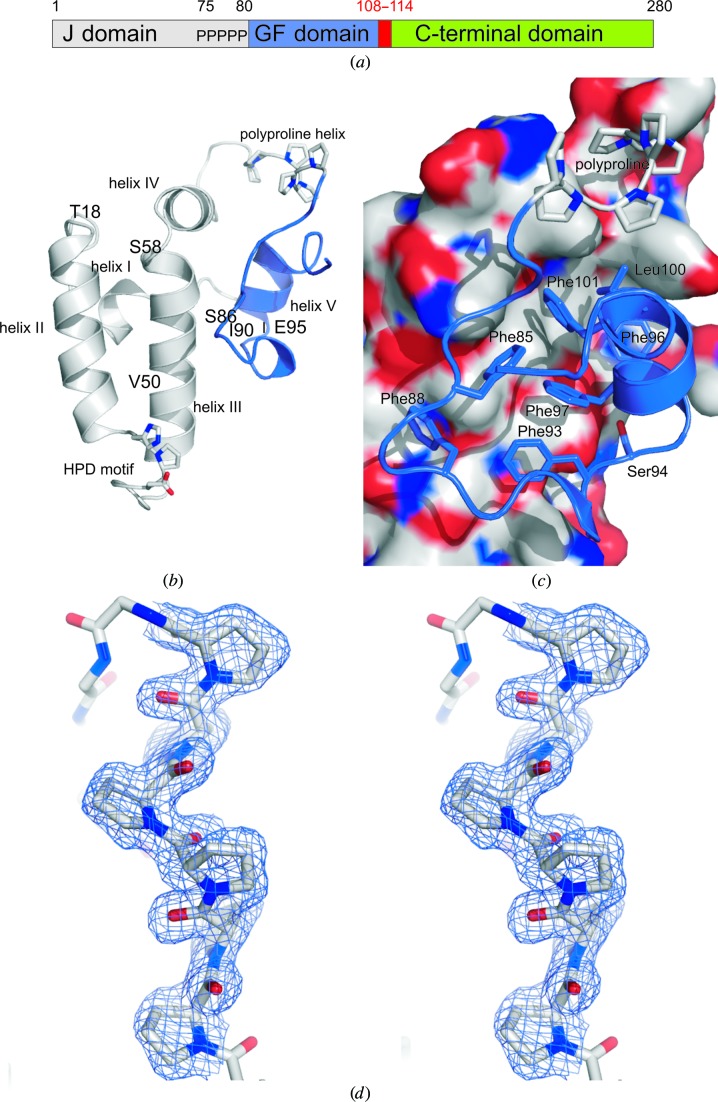
(*a*) DnaJ sequence, showing the J (grey), GF (blue) and C-terminal (green) domains, as well as the polyproline region and the deletion in the DnaJ_Δ108–114_ mutant (red). (*b*) Structure of the isolated J/GF domain. The HPD motif and the polyproline region are shown as sticks. EPR spin-labelling and cross-linking sites (Thr18, Ser58, Ser86 and Glu95) are indicated. (*c*) Interface between the J and GF domains, showing the J-domain surface (coloured by atom type: C, grey; N, blue; O, red) and the GF-domain phenylalanines. All figures use the colour scheme in (*a*). (*d*) Stereo image showing the final refined 2*mF*_o_ − *DF*_c_ electron-density map of the 1.64 Å resolution DnaJ_Tth114_ structure at 1σ. As an example, the polyproline region (residues 75–80) of chain *B* is shown. All molecular-structure figures were prepared using *PyMOL* (DeLano, 2002 ▶)

Based on limited proteolysis of a DnaK–DnaJ–DafA complex (Motohashi *et al.*, 1996 ▶; Klostermeier *et al.*, 1999 ▶), we first designed a construct containing the first 114 amino acids (DnaJ_114_), comprising the J and GF domains, and determined its structure to 1.64 Å resolution (Figs. 1 ▶ and 2 ▶) by radiation-damage-induced phasing (Ramagopal *et al.*, 2005 ▶; Ravelli *et al.*, 2003 ▶; Zwart *et al.*, 2004 ▶) from a mercury(II) chloride derivative. Contrary to expectation, the crystal structure showed an ordered GF domain in the same conformation in all six molecules of the asymmetric unit (Figs. 2 ▶ *b* and 2 ▶ *c*), which are highly similar (the r.m.s. positional differences between C^α^ atoms of any two polypeptide chains range from 0.50 to 0.86 Å for up to 100 C^α^ atoms). After helix IV, the polypeptide chain folds back onto the protein at the Pro_6_ motif, which adopts a polyproline II conformation (Fig. 2 ▶ *d*; Adzhubei & Sternberg, 1993 ▶). After the Pro_6_ motif, the GF domain folds onto one side of the J domain in a spiral, making extensive hydrophobic interactions, mainly *via* the conserved phenylalanine residues. Only two hydrogen bonds are formed between the J and the GF domains: from the Tyr69 hydroxyl group to the side chain of Glu99 and between the side chains of Glu52 and Ser94. In correspondence with the NMR observations for the partially disordered *E. coli* DnaJ GF domain (Pellecchia *et al.*, 1996 ▶), the *T. thermophilus* DnaJ GF domain binds to helix III of the J domain opposite helix II.

### 3.2. The *T. thermophilus* DnaJ_Δ108–114_ variant is biologically active and yields high-quality crystals   

**Figure 3:**
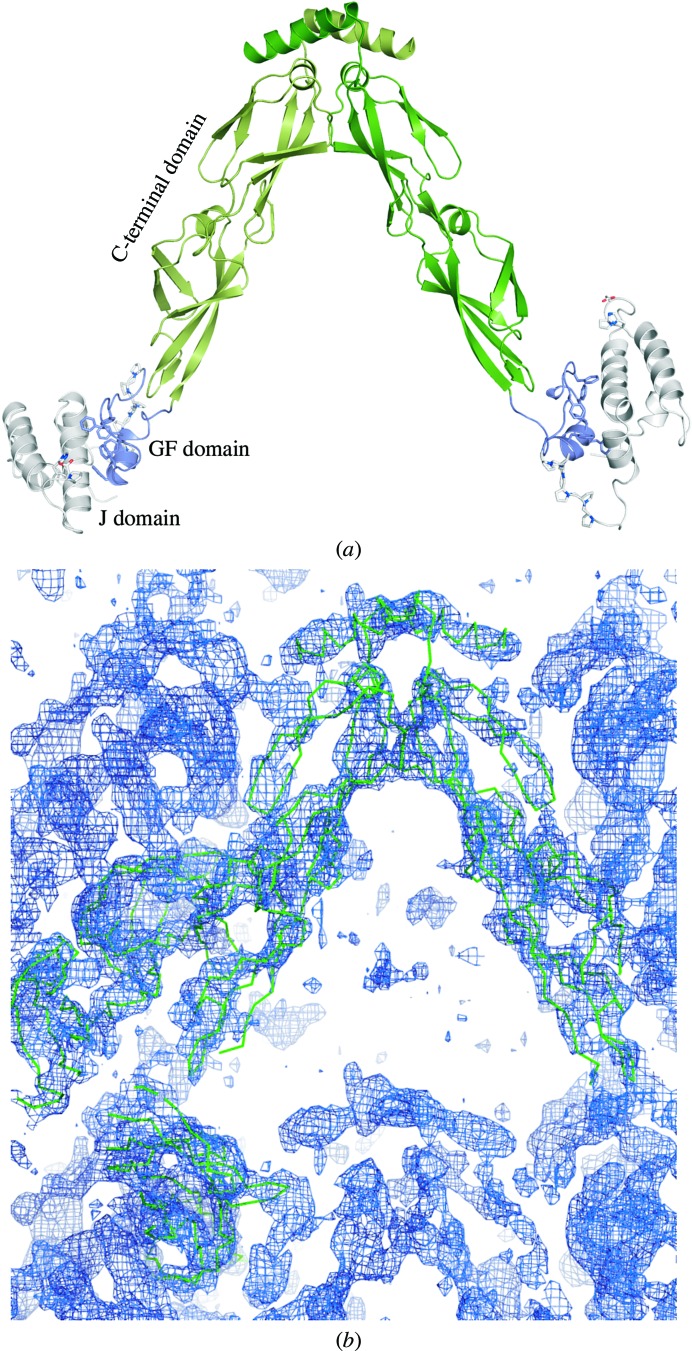
(*a*) Structure of a dimer of DnaJ_Δ108–114_ from the 2.9 Å resolution crystal structure. The relative orientations of the J/GF domains and the C-­terminal domains differ in the four monomers in the asymmetric unit. (*b*) 2*mF*_o_ − *DF*_c_ density at 1.5σ of the 3.8 Å resolution WT data after molecular replacement with the C-terminal domain of DnaJ_Δ108–114_. No density for the N-terminal domains was found and sufficient space for these domains is present in the packing of the molecules.

In the DnaJ_114_ structure, no electron density was observed for residues 108–114 connecting the GF and the C-terminal domains in the complete protein. Therefore, a full-length construct was prepared that lacked the 108–114 stretch (DnaJ_Δ108–114_), which was shown to be functional in refolding assays (see Fig. 6). Crystallization of this variant allowed structure solution to 2.9 Å resolution using SeMet phasing with a construct containing four additional methionines (DnaJ_Δ108–114/4M_), resulting in a structure containing all three domains (Fig. 3 ▶ *a*).

### 3.3. The *T. thermophilus* DnaJ_Δ108–114_ crystal structure also displays an ordered GF domain   

DnaJ_Δ108–114_ dimerizes *via* its C-terminal domains, resulting in a V-shaped molecule with the J/GF domains at the ends of the two stalks (Fig. 1 ▶ *b*) like feet on a pair of legs. The J and GF domains interact tightly through a hydrophobic interface involving six of the seven phenylalanines of the GF domain and helix III of the J domain (Fig. 1 ▶ *c*). The same interaction is seen in all six independent copies of the J/GF domains in the DnaJ_114_ structure. In all four monomers in the asymmetric unit of the DnaJ_Δ108–114_ crystals the orientation of the C-terminal and J/GF domains differs, showing that the Δ108–114 deletion does not restrict their mutual motion. Moreover, the J/GF regions of chains *B* and *D* showed poor electron density, also indicating flexibility.

We also obtained crystals of wild-type DnaJ which diffracted to 3.8 Å resolution after applying a dehydration protocol. Interestingly, in an electron-density map of wild-type DnaJ phased by molecular replacement with DnaJ_Δ108–114_ only the C-terminal domain was observed (Fig. 3 ▶ *b*), indicating a high degree of mobility of the J and GF domains. Indeed, the packing of the wild-type DnaJ crystals leaves sufficient space for the J and GF domains to attain a different orientation with respect to the C-terminal domain in each of the four monomers in the asymmetric unit. Analysis with *DynDom* (Hayward & Lee, 2002 ▶) shows that these orientations vary by as much as 160° and are related to each other by rotations around the region of amino acids 104–113, which connect the J/GF and C-terminal domains. The lack of electron density for the J and GF domains in the wild-type DnaJ structures indicates an even higher degree of freedom there.

### 3.4. Cross-linking shows that DnaJ is flexible in solution   

**Figure 4:**
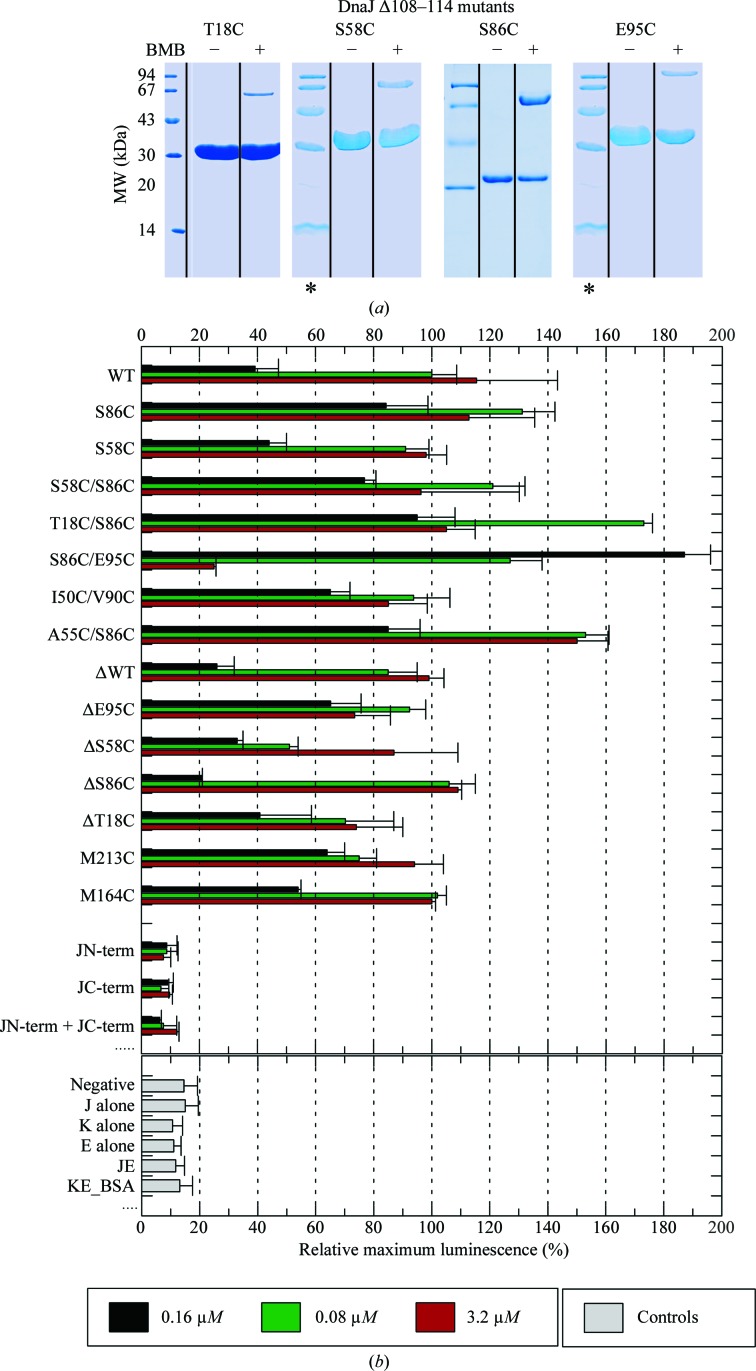
(*a*) Lanes from SDS–PAGE of cysteine mutants of DnaJ_Δ108–114_ without (−) and with (+) cross-linking by BMB after the removal of tetramers by gel filtration (note that the lanes marked * are the same marker lane shown twice for clarity). (*b*) Luciferase-refolding activity of DnaK assisted by DnaJ. DnaJ variants with mutations in the GF domain show particularly strong modulation of refolding activity. Experiments were conducted in triplicate.

Given previous findings of predominantly disordered GF domains (Szyperski *et al.*, 1994 ▶; Pellecchia *et al.*, 1996 ▶; Huang, Ghose *et al.*, 1999 ▶) and our observations of a highly ordered GF domain in crystal structures, we initially surmised an equilibrium between a fully ordered and a more disordered state in solution. To test this, we performed cross-linking with the short (∼11 Å) bifunctional Cys–Cys cross-linker bismaleimidobutane (Fig. 4 ▶ *a*) between single cysteine mutations placed in the J/GF domains of DnaJ_Δ108–114_. This resulted in covalently cross-linked DnaJ_Δ108–114_ dimers, showing that in solution the J/GF domains within a DnaJ_Δ108–114_ dimer can approach each other to within 11 Å, which, given the structure of DnaJ_Δ108–114_, would seem to be possible only when significant disorder exists in solution.

### 3.5. EPR spin-labelling studies show that the GF domain is ordered in solution   

**Figure 5:**
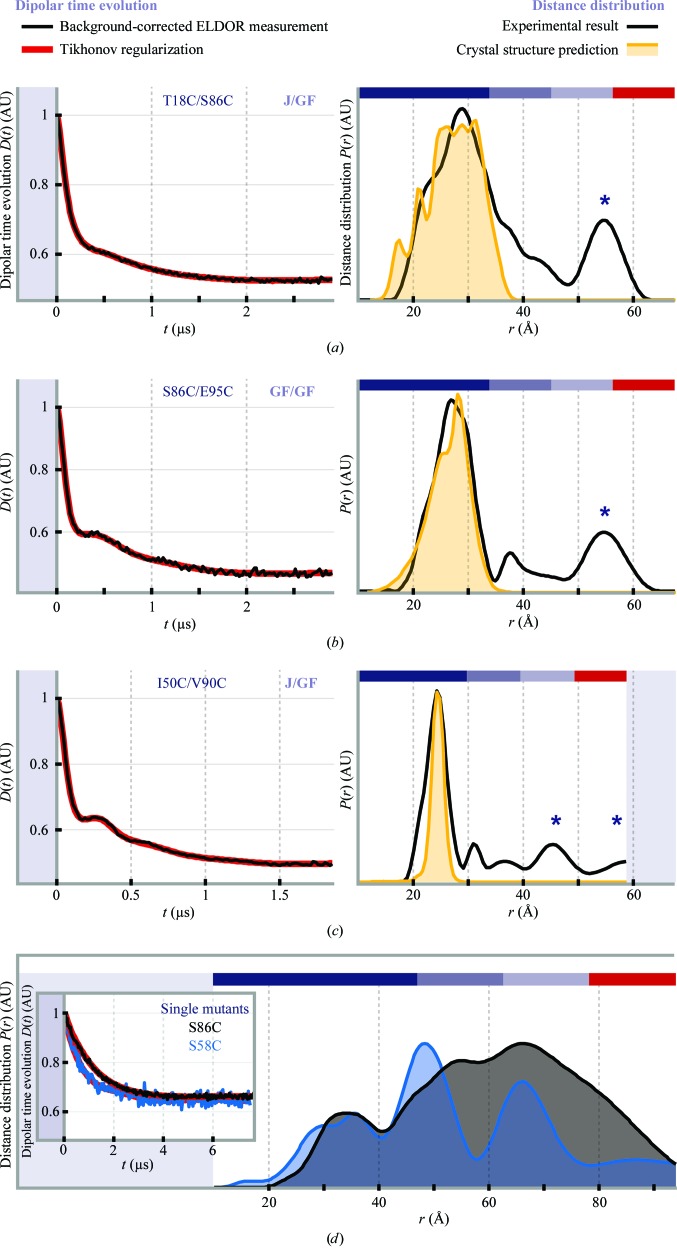
(*a*–*c*) X-band pELDOR measurements of DnaJ double mutants (*a*) T18C/S86C, (*b*) S86C/E95C and (*c*) I50C/V90C: background-corrected time traces and their Tikhonov regularization fits (left; regularization parameter α = 10) and corresponding distance distributions superimposed on their respective rotamer distribution predicted from the crystal structure (right). (*d*) X-band pELDOR measurements of DnaJ single mutants S86C (black) and S58C (blue) using a long time window for measurements of large distances. Dipolar evolution functions with Tikhonov regularization fit (inset; α = 100) and corresponding distance distributions (right). Colour codes above the distributions signify reliability intervals; the reliability of the mean distance of the calculated distribution peaks is shown from dark blue (highest reliability) to red (lowest reliability).

**Figure 6:**
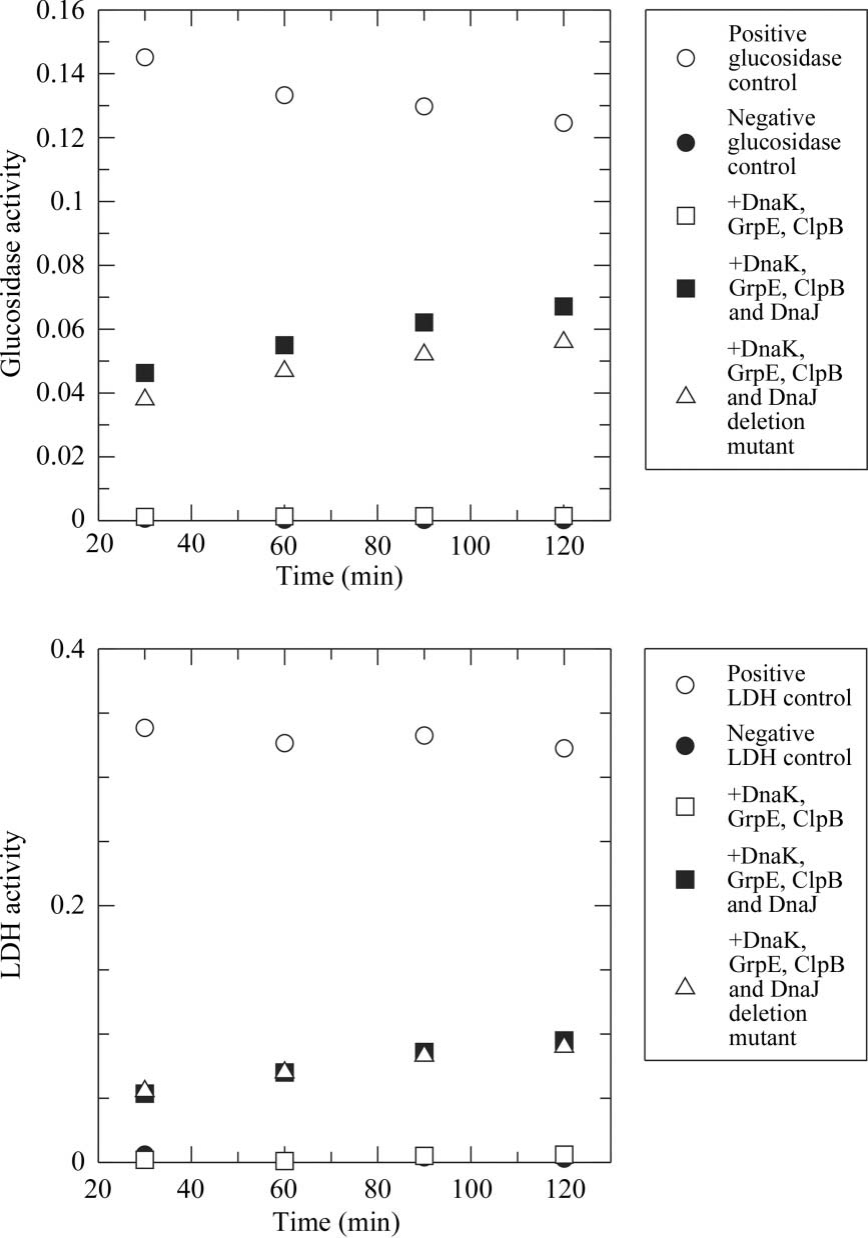
Refolding assays show that SeMet DnaJ_Δ108–114/4M_ is functional.

To check whether this disorder in solution occurs in the GF domain, four-pulse ELDOR (pELDOR) distance measurements (Martin *et al.*, 1998 ▶) were performed on flash-frozen solutions of DnaJ double mutants carrying two spin labels per monomer attached to site-specifically inserted cysteines within the individual J/GF domains (at positions 18 and 86, *i.e.* on the J domain and on the GF domain; at positions 86 and 95, *i.e.* both on the GF domain; and at positions 50 and 90, *i.e.* on the J domain and on the GF domain). Strikingly, these revealed intra­monomer distances (in the 20–30 Å range) which excellently match the distances derived from the crystal structure (Figs. 5 ▶ *a*–5 ▶ *c*). The large modulation depth in the first 250 ns of the time traces governed by these short distances shows that in solution the vast majority of DnaJ molecules adopt the same J/GF structure as observed in the crystal structure. Appendix *B* [App appb] shows additional room-temperature X-band con­tinuous-wave (cw) measurements that further support this notion.

However, additional pELDOR signal contributions occur in the 40–60 Å range (marked by asterisks in Figs. 5 ▶ *a*–5 ▶ *c*) which were not expected from the crystal structure, where intermonomeric distances are only observed at 80 Å and above owing to the pronounced V shape of the dimer. Still, these distances indeed stem from intermonomer dipolar interaction as they are also observed in control experiments on two singly labelled DnaJ variants carrying only one spin label per monomer (Fig. 5 ▶ *d*; labels either at position 58 on the J domain or at position 86 on the GF domain). The broad distance distribution over the whole experimental range shows tails (with low probability down to 20 Å), which is consistent with the cross-linking results and confirms high structural variability in parts of the DnaJ protein backbone in solution.

Given that the individual J/GF sections are structurally rigid at least between Thr18 and Glu95 and that amino acids from Arg118 onwards are clearly visible in the electron-density maps of the wild-type DnaJ crystal structure, any flexibility will most probably be located between Glu95 and Arg118.

### 3.6. Flexibility between the GF and C-terminal domains explains the solution data   

To visualize how much flexibility would be needed to account for the observations described above, we prepared simple geometric models of spin-labelled DnaJ_Δ108–114_ *in silico* from the crystal structure. Starting from nearly static models, in which only rotations around the C^α^—C^β^ bonds of the labelled residues were allowed, we progressively allowed rotations around more and more backbone bonds in the connection between the J/GF and C-terminal domains. For each conformation, a pseudo-energy was calculated from (i) the sum of all C^α^–C^α^ distances smaller than 5 Å and (ii) a term calculated from the EPR distance distribution using the Boltzmann distribution formula. Using these pseudo-energies, Monte Carlo simulations were performed to obtain ensembles of plausible conformations for all models, which were then checked for their consistency with the cross-linking results by checking for the occurrence of conformations in which the cross-linking sites were within 12 Å of each other during a Monte Carlo run of 20 000 trial structures.

The calculations (see Appendix *C* [App appc]) showed that in DnaJ_Δ108–114_, despite the obvious reduction in interdomain flexibility caused by the deletion of seven residues in the J/GF linker, rotation around the backbone bonds of residues 103–110 alone, at the hinge between the J/GF and C-terminal domains, already suffices to explain the cross-linking results.

### 3.7. Mutations in the GF domain modulate the luciferase-refolding activity of DnaK–DnaJ   

The various mutations produced for cross-linking experiments were also evaluated for their ability to assist in luciferase refolding by DnaK. Importantly, not only are all of the mutants employed still active, they also display significant modulation of the luciferase refolding activity, in particular when the mutations are located in the GF domain (Fig. 4 ▶ *b*).

## 4. Discussion   

Given the structural information available previously, the GF domains of DnaJs have frequently been seen as highly dis­ordered flexible structures. The structures and EPR distance distributions of DnaJ presented here necessitate a departure from this view, as they show that in *T. thermophilus* DnaJ the GF domain adopts a highly ordered structure both in the crystal and in solution. Nonetheless, *T. thermophilus* DnaJ is a flexible molecule as shown by cross-linking and EPR studies as well as by the lack of density for the J and GF domains in the DnaJ_wt_ crystal structure. Since the J/GF domains visible in the DnaJ_Δ108–114_ crystal structure each assume a different orientation relative to their respective C-terminal domains, it seems likely that this flexibility is, at least to a large extent, concentrated at the junction between the J/GF domain and the C-terminal domain. Indeed, our very simple molecular modelling studies showed that flexibility in this region alone is sufficient to explain the EPR and cross-linking data. Possibly, the effects that mutations in the GF region have on luciferase refolding are caused by a modulation of this flexibility. In support of this hypothesis, one may compare the *T. thermophilus* and *E. coli* DnaK–DnaJ systems. In *E. coli* DnaJ, which stimulates DnaK ATPase activity strongly, a highly flexible GF domain was observed in NMR studies, although a partial structure was observed (Pellecchia *et al.*, 1996 ▶), and inter­actions with helix III of the J domain (as observed in *T. thermophilus* DnaJ in the current study) were surmised (Huang, Flanagan *et al.*, 1999 ▶). In contrast, in *T. thermophilus* DnaJ, which stimulates DnaK ATPase activity only weakly, the structures presented here indicate a strongly ordered GF domain.

A possible explanation could lie in the structure of the DnaK–DnaJ complex. If, as suggested (Han & Christen, 2003 ▶; Zuiderweg & Ahmad, 2012 ▶), the DnaJ C-terminal domain binds to substrate polypeptide which is also bound to the peptide-binding domain of DnaK, the DnaJ J domain must reach over to another position (Jiang *et al.*, 2007 ▶; Ahmad *et al.*, 2011 ▶; Sousa *et al.*, 2012 ▶; Zuiderweg & Ahmad, 2012 ▶) to effect ATPase stimulation, a situation in which flexibility between the two DnaJ domains would be beneficial and might be modulated for regulatory purposes.

## Supplementary Material

PDB reference: DnaJ, 4j7z

PDB reference: 4j80

## Figures and Tables

**Figure 7:**
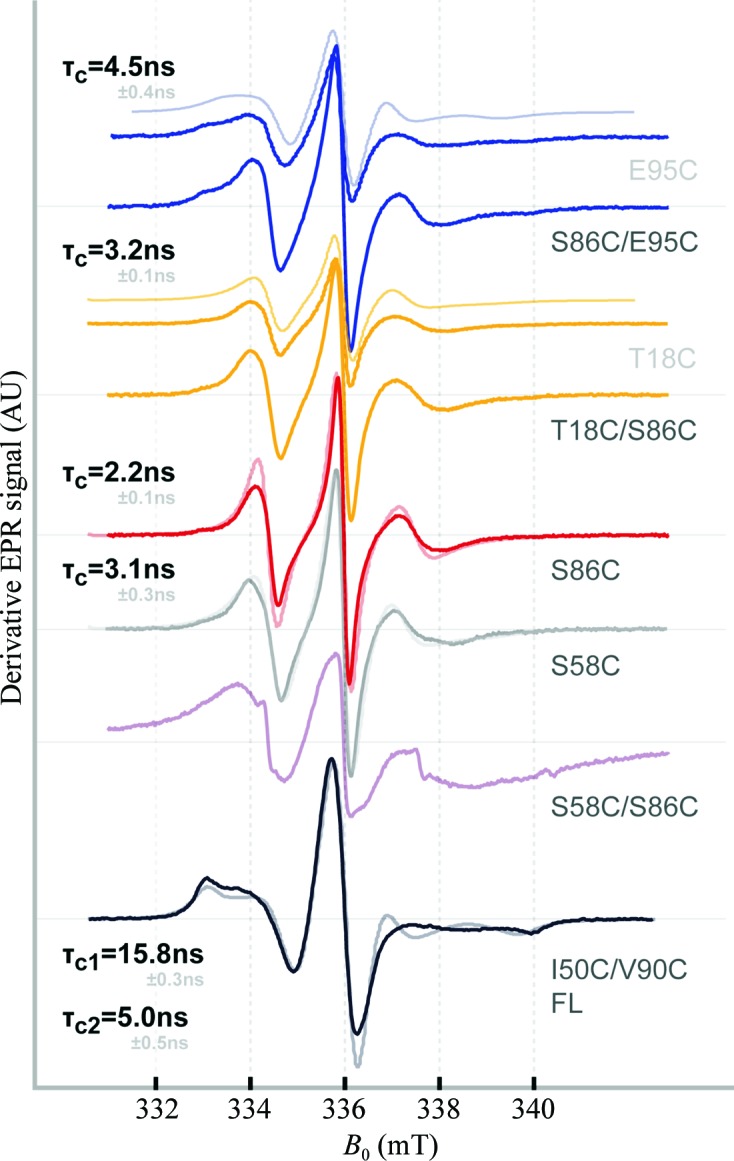
Room-temperature continuous-wave EPR results.

**Figure 8:**
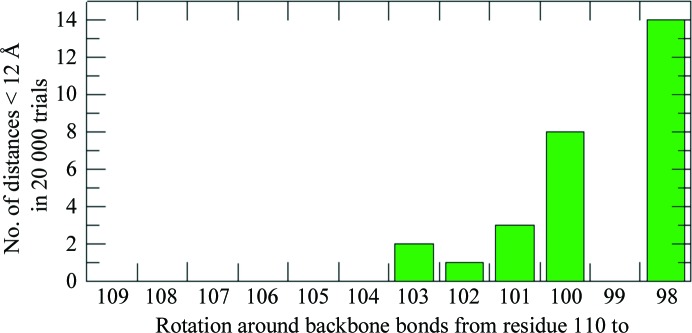
Cross-linking feasibility simulation results.

**Table 1:** Data-collection, phasing and refinement statistics

	DnaJ_114_, 0120 data set, HgCl_2_ derivative	DnaJ_114_, 240360 data set, HgCl_2_ derivative (pseudo-native)	DnaJ_114_, high-resolution data set (4j7z)	DnaJ_108114/4M_, SAD data set	DnaJ_108114/4M_, high-resolution data set (4j80)	DnaJ WT, low resolution
Data collection						
Space group	*P*3_1_		*P*3_1_	*P*2_1_2_1_2	*P*2_1_2_1_2	*P*2_1_2_1_2_1_
Unit-cell parameters ()	*a* = *b* = 84.2, *c* = 71.9		*a* = *b* = 86.0, *c* = 72.7	*a* = 102.8, *b* = 105.3, *c* = 130.6	*a* = 102.1, *b* = 105.0, *c* = 130.0	*a* = 75.1, *b* = 131.6, *c* = 153.1
Resolution ()	402.50 (2.602.50)		201.64 (1.701.64)	803.20 (3.303.20)	202.90 (3.002.90)	403.80 (3.943.80)
*R*_sym_ or *R*_merge_	0.044 (0.197)†	0.041 (0.194)†	0.061 (0.317)	0.086 (0.370)†	0.063 (0.415)	0.104 (0.564)
*I*/(*I*)	17.3 (4.4)	20.6 (4.6)	13.5 (3.4)	19.0 (5.9)	16.2 (3.7)	17.5 (3.6)
Completeness (%)	87.4 (73.4)	85.9 (72.2)	94.5 (82.5)	100 (100)	99.2 (99.8)	99.1 (97.9)
Multiplicity	1.9 (1.9)	2.0 (1.9)	3.1 (2.5)	7.8 (7.8)	4.1 (4.1)	8.0 (7.5)
Refinement						
Resolution ()			201.64		202.90	
No. of reflections			68483		29725	
*R*_work_/*R*_free_			0.214/0.246		0.291/0.328	
No. of atoms						
Protein			4921‡		8460§	
Ligand/ion			18 [glycerol]		0	
Water			341		0	
*B* factors (^2^)						
Protein			35		25	
Ligand/ion			22 [glycerol]			
Water			33			
R.m.s. deviations						
Bond lengths ()			0.010		0.010	
Bond angles ()			1.17		1.15	

† Considering Friedel mates as individual reflections.

‡ Six monomers in the asymmetric unit: chains *A*, *B* and *F*, residues 2101; chain *C*, residues 2104; chain *D*, residues 2107; chain *E*, residues 4101.

§ Two dimers in the asymmetric unit: chains *A*, *B*, *C* and *D* containing residues 2272.

## Appendix A Kinetic measurements

The functionality of SeMet DnaJ_Δ108–114/4M_ was ascertained by refolding assays with lactate dehydrogenase and α-glucosidase as follows: 0.2 µ*M* LDH was denatured for 30 min in a water bath heated to 353 K in denaturation buffer (50 m*M* MOPS/NaOH pH 7.5, 150 m*M* KCl, 10 m*M* MgCl_2_, 5 m*M* ATP, 2 m*M* DTE). For refolding, 192 µl of the denatured sample was added to 8 µl chaperone mixture (final concentrations of 1 µ*M* ClpB, 1.6 µ*M* DnaK, 0.4 µ*M* DnaJ or DnaJΔ_108–114_, 0.2 µ*M* GrpE in denaturation buffer) and incubated for 30, 60, 90 and 120 min. After these refolding periods, 20 µl samples were mixed with 180 µl 50 m*M* Tris–HCl pH 7.5, 50 m*M* KCl, 250 µ*M* NADH, 10 m*M* pyruvate. The turnover of pyruvate and NADH was measured at 298 K by following the decrease of the NADH absorption signal at 340 nm using a VarioSkan MTP reader. The rate was calculated from the linear range of the curves and was plotted against the refolding time (Fig. 6 ▶ *a*).

0.2 µ*M* α-glucosidase in denaturation buffer was denatured for 8 min in a water bath heated to 348 K. For refolding, 192 µl of the denatured sample was added to 8 µl chaperone mixture (final concentrations of 1 µ*M* ClpB, 1.6 µ*M* DnaK, 0.4 µ*M* DnaJ or DnaJ_Δ108–114_, 0.2 µ*M* GrpE in denaturation buffer) and incubated at 328 K for 30, 60, 90 and 120 min. After these refolding periods, 20 µl samples were mixed with 180 µl 50 m*M* potassium phosphate pH 6.8, 2 m*M* PNP-α-GP (*para*-­nitrophenyl-α-d-glucopyranoside), 0.1 mg ml^−1^ BSA in a Corning microtitre plate to yield a final concentration of 20 n*M* α-glucosidase. The turnover of PNP-α-GP was recorded in a VarioSkan MTP reader, following the decrease of absorption at 405 nm at 313 K. The rate was calculated from the linear range of the curve and plotted against the incubation time of refolding (Fig. 6 ▶ *b*).

## Appendix B Room-temperature continuous-wave EPR

All samples examined by pELDOR spectroscopy at cryogenic temperatures (80 and 50 K) were also examined at ambient temperature conditions in an X-band continuous-wave EPR experiment. The width of the observed spectrum can be directly related to a rotational correlation time of the spin-label side chain (*via* the *chili* routine of the *MatLab* package *EasySpin*; Stoll & Schweiger, 2006 ▶) and is thus a probe of the immediate surroundings at the label site. Fig. 7 ▶ shows the recorded spectra as well as estimates for the E95C and T18C single spectra (marked E95C and T18C) in which a 50% proportion of the S86C spectrum was subtracted from the double mutants S86C/E95C and T18C/S86C. For the simulations, the **g** and **A** matrices necessary for the fit were determined from W-band measurements to be **g** = (2.0086, 2.0064, 2.0023) and **A** = (17, 18, 104) MHz (not shown). The rotational correlation times were determined to be 2–5 ns for the single-label spectra and 5 and 15 ns for the I50C/V90C double-label construct (I50C/V90C exhibits especially slow tumbling and strong anisotropy, but both components are still within the same order of magnitude).

In an extensive study, Bordignon *et al.* (2005 ▶) investigated the whole range of labels completely immobilized in a protein to those attached to side chains in unfolded sections. Even the fastest side-chain motion observed for DnaJ S86C is much slower than that observed for a flexible protein backbone. This is further evidence for the GF domain not being unfolded but in a rigid conformation, as observed by pELDOR and crystallography.

## Appendix C Computational modelling

To calculate ensembles of DnaJ conformers, EPR distance distributions *p*(*r*) were converted to pseudo-potential energy surfaces using *E* ^pot^_pseudo_(*r*) = −ln*p*(*r*). To avoid clashes, a penalty term *w* *n*_clashes_ was added to the pseudo-energies, where *w* is a weighting factor and *n*_clashes_ is the number of C^α^ atoms within 3 Å of each other. These pseudo-potential energy surfaces were then used to guide Monte Carlo simulations of DnaJ using specially written *MatLab* scripts, in which only rotations around selected bonds (linker main-chain bonds, see text, and C^α^—C^β^ bonds of labelled residues, which were simulated by tyrosines) were allowed. To check for the possibility of cross-link formation within cysteine mutant dimers, the number of cysteine–cysteine distances smaller than 12 Å was counted in an ensemble of 20 000 structures. Starting with the backbone of residue 110 in DnaJ_Δ108–114_, progressively more backbone bonds were allowed to rotate. When allowing rotations around the backbone bonds of residues 103–110, the first instances of cysteine–cysteine distances below 12 Å were observed. Including more bonds caused a steep increase in the number of distances below 12 Å, as expected (Fig. 8 ▶).
